# Nitroxoline: a potent antimicrobial agent against multidrug resistant Enterobacteriaceae

**DOI:** 10.17179/excli2019-1378

**Published:** 2019-06-26

**Authors:** Rungrot Cherdtrakulkiat, Ratana Lawung, Sunanta Nabu, Srisurang Tantimavanich, Nujarin Sinthupoom, Supaluk Prachayasittikul, Virapong Prachayasittikul

**Affiliations:** 1 Department of Clinical Microbiology and Applied Technology, Faculty of Medical Technology, Mahidol University, Bangkok 10700, Thailand; 2 Center of Data Mining and Biomedical Informatics, Faculty of Medical Technology, Mahidol University, Bangkok 10700, Thailand

**Keywords:** Enterobacteriaceae, multidrug resistance, antimicrobial activity, 8-hydroxyquinoline, nitroxoline

## Abstract

Antimicrobial resistance has become a prime global concern. An ability of the microbes to produce enzymes to destroy antimicrobial drugs is one of the well-known mechanisms underlying the resistance. 8-Hydroxyquinoline (8HQ) and derivatives were reported to exert diverse biological effects such as antimicrobial, antioxidant and antineurodegenerative activities. Herein, 8HQ (1), nitroxoline (NQ, 2) and 7-Br-8HQ (3) were investigated for antimicrobial activity against *Enterobacteriaceae* including extended spectrum β-lactamase (ESBL)-producing and carbapenemase-producing strains as well as the effect of metal ions. These compounds (1-3) displayed the great antimicrobial activity against fifty-eight bacterial isolates of *Escherichia* *coli, Providencia* *rettgeri *and *Klebsiella* *pneumoniae*, in which NQ (2) exerted the highest antimicrobial activity with a MIC_50_ of 42.04 μM (8 µg/mL) and MBC_50_ of 168.28 μM (32 µg/mL). The MIC values of NQ (2) and 7-Br-8HQ (3) were significantly increased in the presence of Cu^2^^+^ and Fe^3+^. This finding reveals that NQ could be an effective compound to be further developed as an antimicrobial agent for combating *Enterobacteriaceae* infections.

## Introduction

*Enterobacteriaceae* is considered to be the largest group of bacteria isolated from clinical specimens. Various genera in this family have been reported such as *Escherichia*, *Klebsiella*, *Enterobacter*, *Citrobacter*,* Serratia*,* Salmonella*, *Shigella*,* Proteus*, *Providencia* and *Morganella*. Additionally, *Plesiomonas*, an oxidase producing genera previously classified as *Vibrionaceae*, has been reclassified as a new member of this family because of its closely related phylogenetic and multilocus sequence typing (MLST) (Janda et al., 2016[10]). In the past, conventional treatment of *Enterobacteriaceae* infection was mainly relied on the use of β-lactam drugs such as penicillins and cephalosporins. β-Lactamase inhibitors such as clavulanic acid, sulbactam and tazobactam were also effectively used in combination with these drugs to combat against some β-lactamase-producing strains i.e., strains producing extended spectrum β-lactamase (ESBL) and AmpC. Besides penicillins and cephalosporins, carbapenems were considered to be the most effective β-lactam drugs against the resistant strains. Unfortunately, the carbapenemase-producing strains have been emerged leading to clinical ineffectiveness of the carbapenems. As a result, urgent discovery and development of novel antimicrobial agents for combating *Enterobacteriaceae* resistance has become a prime concern. Of note, drug repurposing is an effective strategy to discover new clinical applications for the available drugs, which is well known to reduce time and cost of developing new drug candidates (Anighoro et al., 2014[2]; Corsello et al., 2017[7]). As regard, known bioactive compounds or drugs such as 8-hydroxyquinoline (8HQ) and derivatives have drawn considerable attention as potential sources for the drug repurposing.

Previously, 8HQ and derivatives were reported to exhibit diverse bioactivities such as antimicrobial, anti-inflammatory, antioxidant and anticancer activities (Prachayasittikul et al., 2013[18]). The study showed that 8HQ, cloxyquin and clioquinol exerted high potency against gram positive bacteria, whereas gram negative bacteria in the *Enterobacteriaceae* were inhibited by nitroxoline (NQ) and 7-bromo-8HQ (Cherdtrakulkiat et al., 2016[4]). Herein, the antimicrobial activity of 8HQ, nitroxoline and 7-bromo-8HQ were investigated against the *Enterobacteriaceae*, particularly *E. coli* and *Klebsiella* spp., isolated from the routine clinical specimens. In addition, these compounds were also studied against carbapenemase-producing *Providencia rettgeri* NDM-1 as well as the effect of metal ions on the bacterial growth.

## Materials and Methods

### Tested compounds and reagents

**Figure 1:**
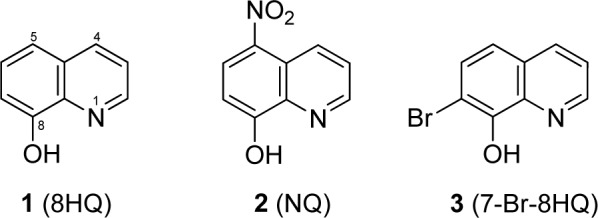
Chemical structures of 8HQ and derivatives (1-3)

8-Hydroxyquinoline (1), 5-nitro-8-hydroxyquinoline (nitroxoline or NQ, 2), and 7-bromo-8-hydroxyquinoline (7-Br-8HQ, 3) were purchased from Sigma-Aldrich, USA. Mueller Hinton broth (MHB) and Mueller Hinton agar (MHA) were obtained from BD Biosciences, USA. Six metal chlorides such as calcium chloride (CaCl_2_), magnesium chloride (MgCl_2_), manganese (II) chloride (MnCl_2_), zinc (II) chloride (ZnCl_2_), copper (II) chloride (CuCl_2_), and iron (III) chloride (FeCl_3_), including dimethyl sulfoxide (DMSO) were supplied with Sigma-Aldrich, USA. Chemical structures of the tested compounds (1-3) are shown in Figure 1(Fig. 1).

### Bacterial isolates and storage

Fifty-six bacterial isolates belonging to the *Enterobacteriaceae* were isolated from clinical specimens in Nakhorn Pathom Hospital, Thailand. All bacterial isolates were confirmed by biochemical test and antimicrobial susceptibility test (disk diffusion method). Bacterial isolates were classified into 4 groups which are 26 isolates of non-ESBL-producing *E. coli*, 27 isolates of ESBL-producing *E. coli*, one isolate of non-ESBL-producing *K*. *pneumoniae*, one isolate of non-ESBL-producing *P. rettgeri *and one isolate of New Delhi metallo-β-lactamase (NDM-1)-producing *P. rettgeri*. In addition, *E. coli* ATCC 25922 and *K. pneumoniae* ATCC 700603 (ESBL-producing strain) were used as the control strains. All bacterial isolates were inoculated in trypticase soy broth (TSB) with 20 % glycerol and were kept at -80 °C for further experiments.

### Antimicrobial activity

#### MIC and MBC of compounds (1-3) against Enterobacteriaceae

Antimicrobial activity of the tested compounds (1-3) was determined using the microdilution method according to the Clinical and Laboratory Standards Institute (CLSI) guideline (CLSI, 2012[5]). Briefly, the tested compound was dissolved in 200 μL DMSO and then mixed thoroughly with the sterile MHB to 1 mL. The solution of compound was two-fold dilution with MHB to make a final concentration ranging from 2-256 µg/mL. Fifty microliters of each diluted compound was pipetted onto the 96-well clear-round bottom microplate. Bacterial isolate was diluted in sterile normal saline, and then was adjusted to 0.5 McFarland standard (1.5 x 10^8^ CFU/mL) using spectro-photometer. Fifty microliters of the adjusted bacterial suspension was mixed to the tested compound in microtiter plate with different concentrations. Each test was performed in triplicates. The microtiter plate was inoculated at 35±2 °C at least 24 hrs. Ampicillin was used as a reference drug. Positive and negative controls were performed by adding bacterial suspension and only MHB in the well, respectively. The minimum inhibitory concentration (MIC) was evaluated as the lowest concentration of tested compound that inhibited the visible growth of bacteria (clear solution). Each clear solution was subcultured on MHA to determine the minimum bactericidal concentration (MBC), which is defined as the lowest concentration of tested compound that bacteria cannot grow on the MHA.

#### Antimicrobial activity of compounds (1-3) against Enterobacteriaceae in the presence of metal ions

The MIC of tested compounds (**1**-**3)** against *Enterobacteriaceae* in the presence of metal ions (Ca^2+^, Mg^2+^, Mn^2+^, Zn^2+^, Cu^2+^, and Fe^3+^) were determined using the microdilution method as previously mentioned (CLSI, 2012[5]). Six metal chlorides (CaCl_2_, MgCl_2_, MnCl_2_, ZnCl_2_, CuCl_2_, and FeCl_3_) were added to the final concentrations ranging from 0.1, 0.5, 1, 10, and 50 mM. The plate was incubated at 35±2 °C at least 24 hrs. Positive control was added bacterial suspension whereas negative control was the compound solution. Each isolate was tested in triplicate and the MIC was measured after the incubation.

## Results

### MIC and MBC of compounds (1-3) against Enterobacteriaceae 

8HQ and derivatives (**1**-**3**) were investigated for antimicrobial potency against fifty-six clinical isolates of the *Enterobacteriaceae*, particularly* E. coli*, using the microdilution method. Ampicillin was tested as the quality control system, and its MIC against *E. coli* ATCC 25922 was shown to be 4 µg/mL as CLSI recommended (2-8 µg/mL) (CLSI, 2017[6]). Results (Table 1(Tab. 1)) showed that compounds (**1**-**3)** exhibited the antimicrobial activity against all bacterial isolates. NQ (**2**) exerted the highest antimicrobial potency (MIC = 21.03-84.14 µM), whereas 8HQ (**1**) and 7-Br-8HQ (**3**) displayed the MIC range of 220.45-881.79 µM and 35.71-142.83 µM, respectively. MIC_50_ and MIC_90_ values of the compounds (**1**-**3**) against both ESBL-producing and non-ESBL-producing *E. coli* isolates were evaluated. NQ (**2**) showed the same MIC_50_ values 42.07 µM in ESBL-producing and non-ESBL-producing *E. coli* isolates. Interestingly, NQ (**2**) also displayed the same MIC value (42.07 µM) against the non-ESBL-producing *P. rettgeri* and *P. rettgeri* NDM-1 strain (carbapenemase-producing strain). In addition, MBC range, MBC_50_ and MBC_90_ values of these compounds (**1**-**3**) are demonstrated in Table 2(Tab. 2). NQ (**2**) showed the MBC_50_ and MBC_90_ against ESBL-producing *E. coli* isolates with values of 168.28 μM and 336.56 µM, respectively. The MBC of compound **2** (673.12 µM) against *K. pneumoniae* ATCC 700603 (ESBL-producing strain) was shown to be higher than the other strains. 8HQ (**1)** and 7-Br-8HQ (**3)** displayed inactive bacteriostatic effect against all *Enterobacteriaceae* isolates (MBC >881.79 µM). 

### MIC of compounds (1-3) against Enterobacteriaceae in the presence of metal ions

Effects of metal ions (Ca^2+^, Mg^2+^, Mn^2+^, Zn^2+^, Cu^2+^, and Fe^3+^) on the antimicrobial activity of 8HQ and derivatives (1-3) against *Enterobacteriaceae* isolates were investigated at various concentrations (0.1-50 mM). The inoculation of tested compounds and all *Enterobacteriaceae* isolates without metal ions were used as the control. The antimicrobial activity (Table 3(Tab. 3)) of compounds (1-3) was reduced in the presence of Mn^2+^, Zn^2+^, Cu^2+^ and Fe^3+^ whereas Ca^2+^ and Mg^2+^ showed no effect on their activities. In the presence of 0.1 mM Mn^2+^, the MICs of all compounds against *Enterobacteriaceae* isolates were not different from the control, except for 8HQ (1) against *E. coli* ATCC 25922 and *E. coli* CTX-M1 (4-fold increasing of MIC). MIC values of compound 1 against all bacterial isolates were increased when adding Mn^2+^, Zn^2+^, Cu^2+^ and Fe^3+^, whereas MIC values of compounds 2 and 3 were significantly increased only in the presence of Cu^2+^ and Fe^3+^. At 0.5 mM Cu^2+^ and 0.5 mM Fe^3+^, MICs of compounds 2 and 3 against *Enterobacteriaceae* isolates were shown to be increased at least 8-fold compared with the control. When the concentrations of metal ions were 1, 10 and 50 mM, the MIC of tested compounds (1-3) against all bacterial strains were not significantly different. Therefore, Table 3(Tab. 3) demonstrated only the effect of metal ions at the concentrations of 0.1, 0.5 and 1 mM. 

## Discussion

The present study showed that NQ (2) exerted the highest antimicrobial potency (MIC = 21.03-84.14 μM) against all *Enterobacteriaceae* strains, including carbapenemase-producing *P. rettgeri* which can resist to the most effective drug in β-lactam groups. The gap between MIC_50 _and MBC_50 _values of NQ (2) is greater than two-fold dilutions indicating that compound 2 exerted bacteriostatic effect toward *E. coli*. Interestingly, most of 8HQ and derivatives exhibited the antimicrobial activity against the non-ESBL-producing strains and ESBL-producing strains with the same MIC values, which may be due to an inability of the produced enzyme to affect the compounds. In addition, 8HQ showed the lowest antimicrobial activity (MIC = 220.45-881.79 μM), although it was reported as the most active compound against gram positive bacteria such as *Staphylococcus aureus, Listeria monocytogenes *and* Bacillus subtilis* (Cherdtrakulkiat et al., 2016[4]).

NQ (**2**) and 7-Br-8HQ (**3**) with lipophilic substitutions at 5-position (NO_2_) and 7-position (Br), respectively, might enhance better absorption through the lipopolysaccharide (LPS) in the outer membrane of gram negative bacteria leading to improve antimicrobial activity compared with the parent 8HQ (**1**). However, these compounds (**1**-**3**) showed the highest MIC values when tested with *Klebsiella pneumoniae*, because these bacteria can produce the capsule layer which protected themselves from toxic substances (Amako et al., 1988[1]). Moreover, *K. pneumoniae* has many mechanisms to impair uptake and avoid contact with the antimicrobial drugs including to decrease permeability of outer membrane or to increase efflux pump. (Bi et al., 2017[3]). 

*E. coli*, *K*.* pneumoniae*, and* P. rettgeri* are usually isolated from specimens in the routine clinical microbiology laboratories. Most of them have been identified as opportunistic pathogens that are commonly responsible for urinary tract infection (UTI) (Sharma et al., 2017[19]; Wagenlehner et al., 2014[20]). The most causative pathogen of UTI has been reported such as *E. coli* and occasionally *K. pneumoniae* (Wagenlehner et al., 2014[20]). Besides the UTI, *E. coli*, *K*.* pneumoniae* and *P. rettgeri* cause a wide variety of infections. *E. coli* can cause gastroenteritis, neonatal meningitis, hemorrhagic colitis, and pneumonia (Kaper et al., 2004[11]). *K. pneumoniae* also causes severe pneumonia, upper respiratory tract infection, wound infection, meningitis, and septicemia (Khan et al., 2015[12]). *P. rettgeri* has been partly reported as a causative pathogen of neonatal sepsis (Sharma et al., 2017[19]).

In the past, the third generation cephalosporins such as ceftriaxone, cefotaxime and ceftazidime were used for treatment of the *Enterobacteriaceae* infections. However, the *Enterobacteriaceae* strains have produced the ESBL enzyme to hydrolyze β-lactam ring, the active part, of these drugs. Therefore, the carbapenems have been used for the treatment. Recently, the carbapenemase-producing *Enterobacteriaceae* (CPE) has been emerged worldwide. Unfortunately, carbapenems such as ertapenem, meropenem, imipenem and doripenem become ineffective drugs for such treatment. Although β-lactam drugs are prohibited, the other classes of drugs such as aminoglycosides (amikacin), fluoro-quinolones (ciprofloxacin, levofloxacin), tetracyclines (doxycycline) including tigecycline and colistin, are available as alternative drugs for treating resistant *Enterobacteriaceae* isolates.

In the presence of metal ions (Pelletier et al., 1995[16]; Prachayasittikul et al., 2013[18]), an antagonistic effect was noted for the compounds (**1**-**3**), in which 8HQ (**1**) showed the reduction of antimicrobial activity when adding Mn^2+^, Zn^2+^, Cu^2+^, and Fe^3+ ^ at any concentrations (0.1-50 mM), whereas the activities of NQ (**2**) and 7-Br-8HQ (**3**) were reduced only in the presence of Cu^2+^, and Fe^3+^. These results supported that the 8HQ and derivatives (**1**-**3**) have the ability to chelate metal ions and reduce their antimicrobial functions (Porcheron et al., 2013[17]). At the same time, the metal ions might be served as the cofactors of bacteria to survive and to resist these compounds (Palmer and Skaar, 2016[15]). 

Recently, 8HQ derivatives have been reported as candidates to be developed as anti-gonorrhoeal agents including a multidrug resistant strain (ceftriaxone and cefixime resistances) (Lawung et al., 2018[13]). In addition, *in vivo* study of 8HQ and derivatives was documented as antileishmania agents in BALB/C mice (Duarte et al., 2016[9][8]).

Currently, the emergence of antimicrobial resistant bacteria is a prime global problem, especially the *Enterobacteriaceae*. Therefore, the drug discovery and development for new potent compounds is an urgent issue to combat multidrug-resistant *Enterobacteriaceae*. This finding reveals that the NQ (**2**), the FDA approved drug (Lazovic et al., 2015[14]), is a potential candidate to be further developed as antimicrobial agent for treatment of *Enterobacteriaceae* infections. Additionally, the effect of metal ions may also be noteworthy for future drug development.

## Acknowledgement

We gratefully acknowledge the support (for S.N. as a postdoctoral fellow) by Office of the Higher Education Commission, Mahidol University under the National Research Universities Initiative and Annual Government Grant under Mahidol University (2562-2563 B.E.), Thailand. 

## Conflict of interest

The authors declare no conflict of interest.

## Figures and Tables

**Table 1:**
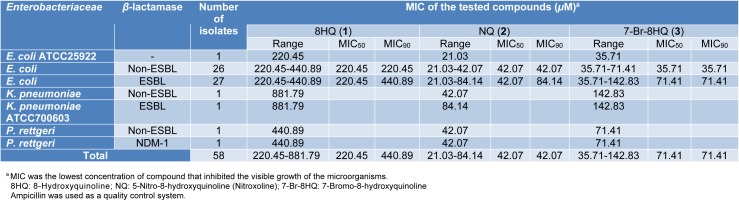
MIC of 8HQ and derivatives (1-3) against *Enterobacteriaceae*

**Table 2:**
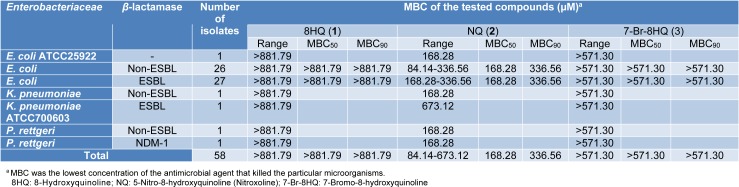
MBC of 8HQ and derivatives (1-3) against *Enterobacteriaceae*

**Table 3:**
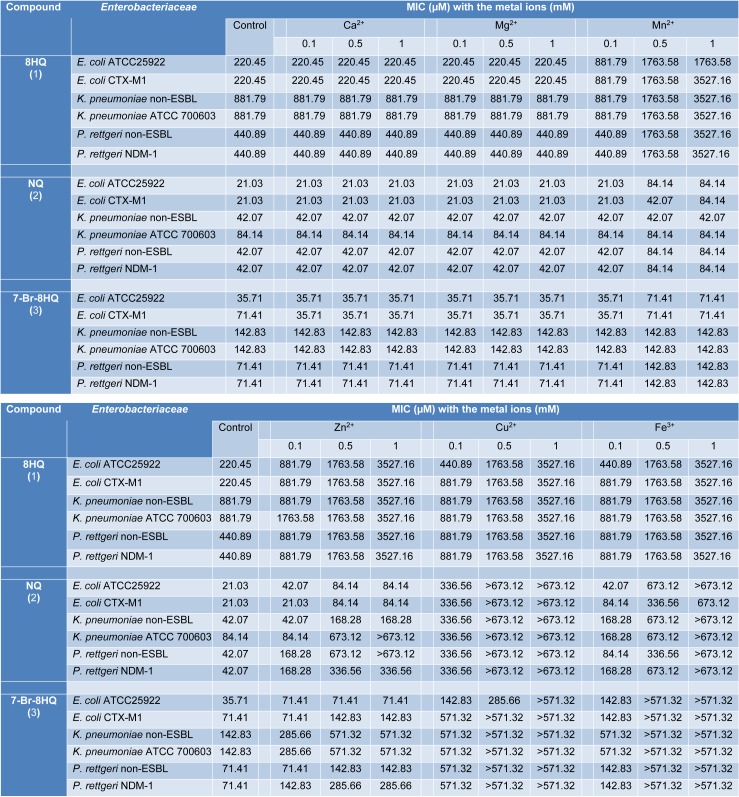
MIC of compounds (1-3) against *Enterobacteriaceae* in the presence of metal ions
